# An extremely rare case of tubo-ovarian abscesses involving *corynebacterium striatum* as causative agent

**DOI:** 10.1186/s12879-016-1860-0

**Published:** 2016-09-29

**Authors:** Tetsuya Yamamoto, Tsuneaki Kenzaka, Shimpei Mizuki, Yuki Nakashima, Houu Kou, Motoyoshi Maruo, Hozuka Akita

**Affiliations:** 1 Department of Internal Medicine, Hyogo Prefectural Kaibara Hospital, Tamba, Japan; 2 Division of Community Medicine and Career Development, Kobe University Graduate School of Medicine, 2-1-5, Arata-cho, Hyogo-ku, Kobe, Hyogo 652-0032 Japan; 3 Department of Obstetrics and Gynecology, Hyogo Prefectural Kaibara Hospital, Tamba, Japan

**Keywords:** *Corynebacterium striatum*, Tubo-ovarian abscesses, Psoriasis vulgaris, Upper reproductive tract infection

## Abstract

**Background:**

We present an extremely rare case of tubo-ovarian abscesses involving *Corynebacterium striatum* (*C. striatum*) as causative agent in a 53-year-old woman.

**Case presentation:**

The patient presented with stomach pain, chills, and nausea. Her medical history included poorly controlled psoriasis vulgaris and diabetes. Laboratory and imaging findings led to diagnosis of septic shock due to tubo-ovarian abscesses. She was treated with antibiotic therapy and surgery to remove the left adnexa. Various cultures detected *Prevotella spp.* and *C. striatum*. We concluded that *C. striatum* from skin contaminated by psoriasis vulgaris had caused the tubo-ovarian abscesses by way of ascending infection.

**Conclusions:**

This may be the first known case of tubo-ovarian abscesses due to *C. striatum*. In patients whose skin has been weakened by psoriasis vulgaris or other infections, *Corynebacterium* should be considered as causative microorganisms, and antibiotic therapy including vancomycin should be administered.

## Background

Tubo-ovarian abscesses are inflammatory masses involving the fallopian tubes and ovaries as well as adjacent pelvic internal organs, and usually arise as lower genital tract infections that ascend and seed the upper reproductive tract (uterine body, fallopian tubes, ovaries) in women of reproductive age [1]. Tubo-ovarian abscesses usually occur as a complication of pelvic inflammatory disease. The infection typically involves multiple types of bacteria, including vaginal flora and intestinal bacteria [2, 3].

Here, we present an extremely rare case of tubo-ovarian abscesses involving *Corynebacterium striatum* (*C. striatum*) as causative agent.

## Case presentation

**Fig. 1:**
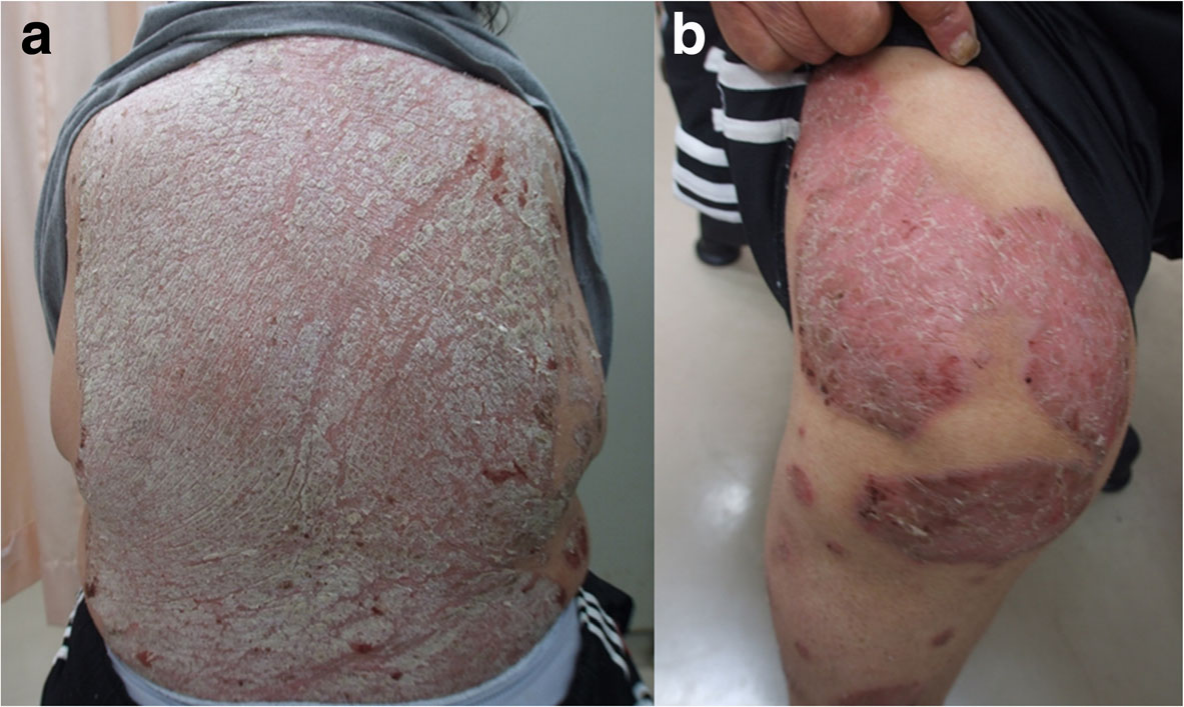
Photographs of the patient’s back (**a**) and right knee (**b**) 1 year prior to admission, showing poorly controlled psoriasis vulgaris

A 53-year-old woman presented to the emergency department for evaluation of abdominal pain, chills, and nausea. Her medical history was notable for psoriasis vulgaris, diagnosed at age 42, for which she had received intermittent treatment, but had not received any treatment in the preceding 6 months (Fig. 1). She also had high blood pressure, received a diagnosis of type 2 diabetes at age 30, and underwent percutaneous transluminal coronary angioplasty for unstable angina at age 52. Her gynecologic history was notable for one Caesarian section. She underwent menopause at age 48, and was found to have a right ovarian cyst at age 52. She had no recent history of sexual intercourse for at least 5 years.

The patient had felt abdominal bloating and pain for 3 days prior to presentation. Two days before presentation, the pain had moved to the lower left part of the abdomen, where it was localized. On the day of presentation, she had felt chills and nausea and requested transportation by ambulance for emergency treatment.

**Fig. 2:**
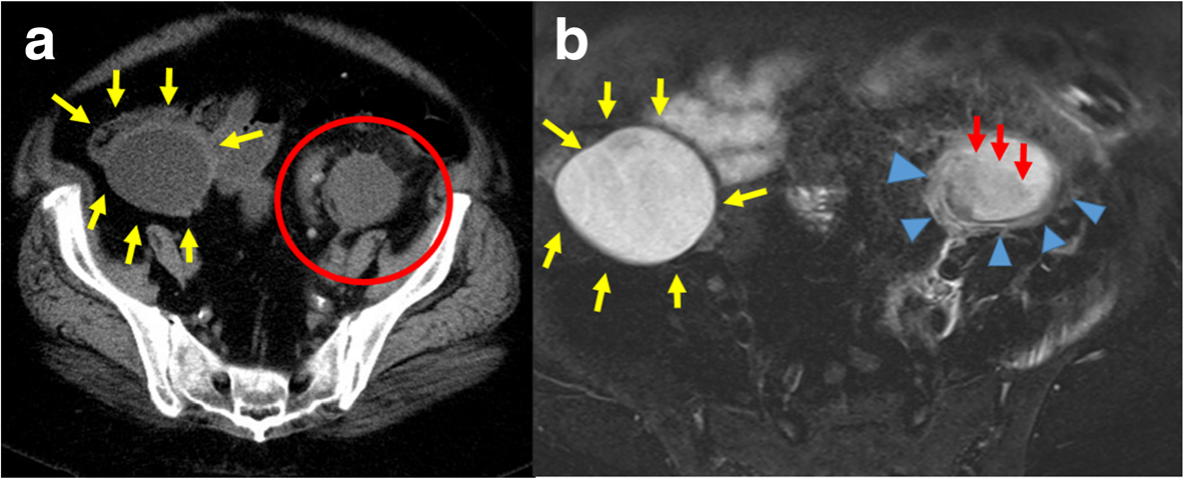
Computed tomographic and magnetic resonance imaging findings on admission. **a** Computed tomography scan of the abdomen and pelvis, showing a 38 × 44-mm tumor surrounded by dense adipose tissue (*red circle*) in the left adnexa. The previously noted right ovarian abscess also is seen (*yellow arrows*). **b** Coronal T2-weighted contrast-enhanced magnetic resonance image of the pelvis, showing edematous change (*red arrows*) surrounded by liquid formation (*blue arrowheads*) in the left adnexa. The previously noted right ovarian abscess also is seen (*yellow arrows*)

On presentation, the patient’s body mass index was 38.2 kg/m^2^. Her vital signs were as follows: body temperature, 40.1 °C; blood pressure, 98/65 mmHg; pulse, 108 beats/min; respiratory rate, 16 breaths/min; and oxygen saturation, 98 % on room air. On physical examination, she was tender to palpation throughout the left abdomen, but no clear peritoneal irritation symptoms were noted. Poorly controlled psoriasis vulgaris also was found, and because she had not bathed for more than 10 days, the skin was covered by filthy sebum secondary to poor hygiene. Laboratory findings were as follows: leukocyte count, 5480/μL; neutrophils, 92 %; C-reactive protein level, 20.3 mg/dL; procalcitonin level, 2.41 ng/mL; blood urea nitrogen level, 22.2 mg/dL; and creatinine level, 1.99 mg/dL (Table 1). Computed tomography of the abdomen and pelvis revealed a 38 × 44 × 68-mm mass surrounded by dense adipose tissue in the left adnexa, which raised suspicion of infection (Fig. 2a). T2-weighted contrast-enhanced magnetic resonance imaging of the pelvis revealed edematous change surrounded by liquid formation in the left adnexa, which raised suspicion of ovarian abscess (Fig. 2b).

**Table 1:** Laboratory data on admission

Parameter	Recorded value	Standard value
White blood cell count	5480/μL	4500–7500/μL
Neutrophils	92 %	
Hemoglobin	10.6 g/dL	11.3–15.2 g/dL
Hematocrit	33.2 %	36–45 %
Platelet count	17.6 × 10^4^/μL	13–35 × 10^4^/μL
International normalized ratio	1.08	0.80–1.20
Activated partial thromboplastin time	28.3 s	26.9–38.1 s
Fibrin degradation products	15.0 μg/mL	2.0–8.0 μg/mL
C-reactive protein	20.3 mg/dL	≤0.14 mg/dL
Procalcitonin	2.41 ng/mL	≤0.05 ng/mL
Total protein	7.5 g/dL	6.9–8.4 g/dL
Albumin	3.1 g/dL	3.9–5.1 g/dL
Total bilirubin	0.7 mg/dL	0.2–1.2 mg/dL
Aspartate aminotransferase	16 U/L	11–30 U/L
Alanine aminotransferase	16 U/L	4–30 U/L
Lactate dehydrogenase	173 U/L	109–216 U/L
Creatine phosphokinase	20 U/L	40–150 U/L
Blood urea nitrogen	22.2 mg/dL	8–20 mg/dL
Creatinine	1.99 mg/dL	0.63–1.03 mg/dL
Sodium	134 mEq/L	136–148 mEq/L
Potassium	5.0 mEq/L	3.6–5.0 mEq/L
Glucose	251 mg/dL	70–109 mg/dL
Hemoglobin A1c	9.2 %	<6.5 %
pH	7.376	7.350–7.450
Partial pressure of carbon dioxide	40.1 mmHg	35.0–45.0 mmHg
Bicarbonate ion	23.0 mEq/L	23.0–28.0 mEq/L
Lactic acid	5.10 mmol/L	0.44–1.78 mmol/L
Anion gap	18.3 mEq/L	10.0–14.0 mEq/L

At the time of admission, the patient was in a preshock state with findings of peripheral circulatory insufficiency and renal dysfunction, and was admitted to the intensive care unit. Based on laboratory and imaging findings, she was diagnosed with severe sepsis secondary to tubo-ovarian abscess. She was started on empiric antibiotic therapy comprising of meropenem 1 g every 12 h, vancomycin 1 g every 24 h, and minocycline 100 mg every 12 h. Although there was no evidence of rupture of the tubo-ovarian abscess on imaging, her clinical status deteriorated acutely due to progression of sepsis. During hospitalization, her circulatory dynamics were disrupted (she had hypotension and tachycardia) and addition of a vasopressor became necessary. Urine volume also declined and renal function worsened, such that maintaining blood pressure became increasingly difficult even with a vasopressor. On the second day of hospitalization, given that the patient was demonstrating signs of septic shock, emergency abdominal surgery was performed to control the infection source.

**Fig. 3:**
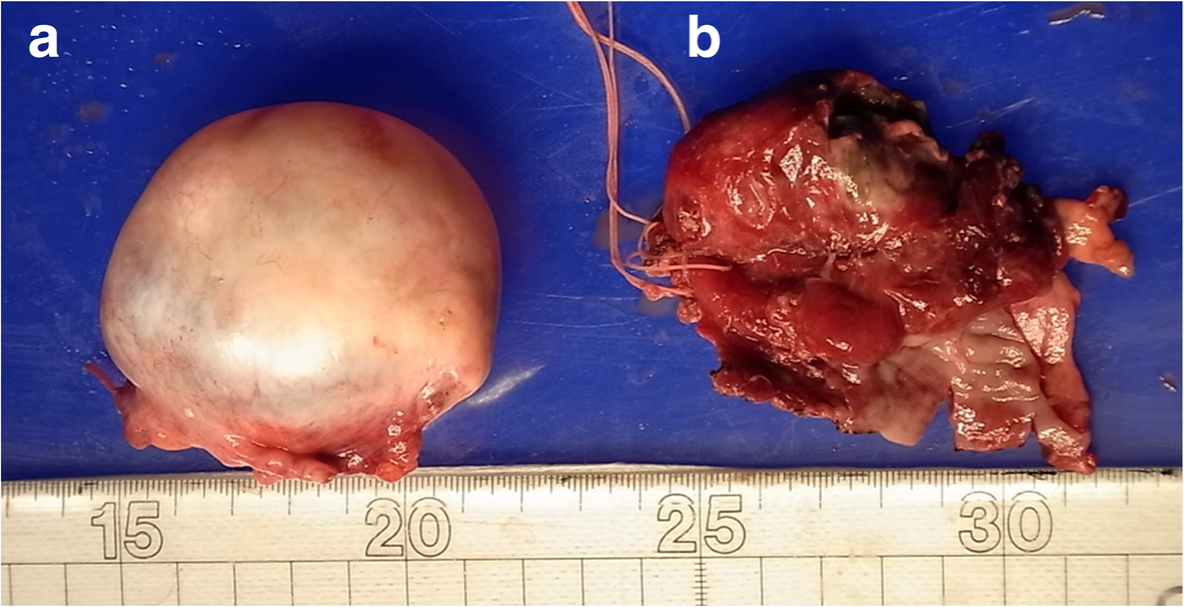
Gross findings of the surgical specimens. **a** Ovarian abscess. **b** Ovarian abscess section surface

An abscess had formed from the left ovary, entangling the fallopian tube; thus, resection of the left adnexa was performed. While no evidence of rupture was found, there were significant adhesions in the large intestine around the left adnexa. Infection lesions were found in the left ovarian parenchyma extending to the surrounding organs, including the fimbriae of the fallopian tube, with diffuse infiltration of inflammatory cells of neutrophil bodies across a wide range, and randomly spaced formation of large and small abscesses. No malignant findings were noted. Gross findings of the excised specimens are shown in Fig. 3.

Blood cultures detected *Prevotella spp.*, ovarian abscess cultures identified *Prevotella* and *C. striatum*, and vaginal and perineal skin cultures also revealed *C. striatum. C. striatum* was identified by using the API Coryne identification panel (bioMerieux, Marcy l’Etoile, France). Vaginal and ovarian abscess cultures were negative for *Chlamydia trachomatis*, *Neisseria gonorrhoeae*, and other microbes. Also, *Chlamydia trachomatis* and *Neisseria gonorrhoeae* were negative according to polymerase chain reaction. Based on these culture results, meropenem was changed to ampicillin/sulbactam 3 g every 8 h, and vancomycin and minocycline were continued.

**Fig. 4:**
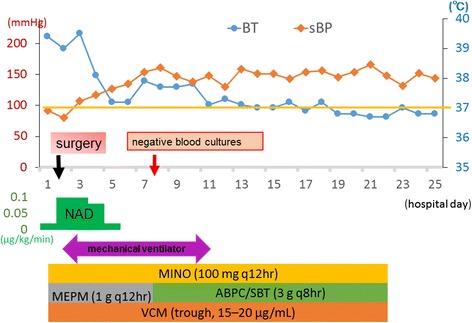
Summary of the patient’s clinical course from admission through day 25. Although her blood pressure decreased before surgery, her hemodynamics slowly stabilized after surgery. On day 15, she was moved from the high care unit to a general patient wing. In accordance with the blood culture results, treatment proceeded with ampicillin/sulbactam and vancomycin. Blood cultures on day 8 were negative. Abbreviations: ABPC/SBT, ampicillin/sulbactam; BT, body temperature; MEPM, meropenem; MINO, minocycline; NAD, noradrenalin; sBP, systolic blood pressure; VCM, vancomycin

After surgery, the patient’s hemodynamics slowly stabilized, urine volume gradually increased, and renal function improved. Blood cultures on the eighth day were negative; thus, antibiotic therapy was implemented for 14 days. On the 15th day, she was moved from the intensive care unit to a general ward. A summary of the patient’s clinical course is shown in Fig. 4. She was discharged 45 days after admission. Currently, it has been 6 months since her discharge from the hospital, and she has been in good health.

## Discussion

We presented a case of tubo-ovarian abscess due to *C. striatum* and *Prevotella*. Tubo-ovarian abscess due to *C. striatum* is extremely rare. We concluded that *C. striatum* from skin contaminated by psoriasis vulgaris had caused the tubo-ovarian abscesses by way of ascending infection. Although vaginal cultures were negative for anaerobic bacteria, we considered that ascending infection also occurred due to *Prevotella spp.*

Infection paths of tubo-ovarian abscesses include ascending infection from the vagina; hematogenous, lymphatic, or other descending infection; or spread of intra-abdominal infection after surgery [4]. Common infectious bacteria include *Escherichia coli*, aerobic streptococci, *Bacteroides fragilis*, *Prevotella*, and other anaerobes, such as *Peptostreptococcus* [2], while scattered case reports also mention *Candida* [5], *Pasteurella multocida* [6], *Salmonella* [7], and *Streptococcus pneumoniae* [8]. There are also rare reports of immunocompromised cases due to tuberculosis [9]. To the best of our knowledge, this is the first reported case of tubo-ovarian abscess and upper reproductive tract infection due to *Corynebacterium*.

In this case, *C. striatum* was detected in cultures of the ovarian abscesses, vaginal secretions, and perineal skin. In addition, weakening of the skin barrier due to psoriasis vulgaris, skin contamination secondary to poor hygiene, and contamination around the perineum were strongly evident. Based on these findings, we concluded that *C. striatum* from skin resident microbiota had caused the tubo-ovarian abscesses by way of ascending infection.

*Corynebacterium* bacteria are part of the normal flora of the skin and upper respiratory tract, and are usually not identified in specimens from sputum or the nasal cavities. In addition, because *Corynebacterium* is often regarded as contamination in such specimens, drug sensitivity tests are rarely performed. In addition, even when detected in blood cultures, if the patient’s clinical condition is poor, some physicians might conclude that it is due to contamination when the specimens were drawn. However, *C. striatum* has attracted attention for its involvement in respiratory infections, wound infections, infectious endocarditis, and urinary tract infections as an opportunistic infection or microbial-substitution disease [10, 11].

Commonly, tubo-ovarian abscess is caused by pelvic inflammatory disease, and a treatment regimen covering the above-mentioned high-frequency causal microorganisms and sexually transmitted pathogens (including *Neisseria gonorrhoeae* and *C. trachomatis*, even though these bacteria are rarely isolated from tubo-ovarian abscesses) is recommended. Specifically, any combined use of cefoxitin and doxycycline; cefotetan and doxycycline; or ampicillin/sulbactam and doxycycline is recommended as a first-line treatment [2, 12–14]. Broad-spectrum antibiotics should be used. After the culture results were received, we continued to use three antimicrobial drugs for the pelvic inflammatory disease and anaerobic bacteria that were not cultured. Approximately 70 % of cases can be treated effectively with only antibiotics, i.e., resolved without surgery [2]. However, as shown in Table 2, consideration of surgery is necessary for larger abscesses or with rupture of the abscess.

**Table 2:** Recommendations of surgery for tubo-ovarian abscesses

Emergency surgery [15]
✓ Abscess rupture suspected
✓ Signs of sepsis, such as low blood pressure, tachycardia, or tachypnea
✓ Acute abdominal pain
Surgery or drainage [15, 16]
✓ Abscess diameter >8 cm
✓ No therapeutic reaction 48 h after administration of antibiotics
Consideration of surgery [17]
✓ Postmenopausal patient

In the present case, the diagnosis was tubo-ovarian abscesses and severe sepsis. Given the severity of the disease, broad-spectrum antibiotics were used, including meropenem, minocycline, and vancomycin, though they are not typically first-line treatments. This regimen covered both *Prevotella* and *C. striatum*. When the patient’s condition deteriorated due to septic shock, emergency surgery also was performed. The above-mentioned recommended first-line therapy did not successfully treat the *C. striatum*. Therefore, in cases with psoriasis vulgaris or other skin diseases in which the skin barrier is weakened or skin contamination is strong, we believe that an antibiotic regimen including vancomycin is necessary out of consideration for *Corynebacterium* or methicillin-resistant *Staphylococcus aureus*. Finally, appropriate timing of surgery is essential for successful treatment of tubo-ovarian abscesses.

## Conclusion

This may be the first known case of tubo-ovarian abscesses due to *C. striatum*. In patients whose skin has been weakened by psoriasis vulgaris or other infections, *Corynebacterium*, which is normally considered as a skin contaminant, should be considered as a causative microorganism, and antibiotic therapy including vancomycin should be administered.

## Abbreviations

*C. striatum*: *Corynebacterium striatum*
